# Outbreak of *Escherichia coli* O104:H4 Infections Associated with Sprout Consumption — Europe and North America, May–July 2011

**Published:** 2013-12-20

**Authors:** Catherine Foley, Emily Harvey, Sally A. Bidol, Tiffany Henderson, Rebecca Njord, Traci DeSalvo, Thomas Haupt, Adamma Mba-Jonas, Chris Bailey, Cheryl Bopp, Stacey A. Bosch, Peter Gerner-Smidt, Rajal K. Mody, Thai-An Nguyen, Nancy Strockbine, Robert V. Tauxe

**Affiliations:** Arizona Dept of Health Svcs; Massachusetts Dept of Public Health; Michigan Dept of Community Health; North Carolina Dept of Health and Human Svcs; Wisconsin Div of Public Health; Dept of Epidemiology, Center for Biologics Evaluation and Research, Food and Drug Administration; Div of Foodborne, Waterborne, and Environmental Diseases, National Center for Emerging and Zoonotic Infectious Diseases, CDC

In May 2011, public health authorities in Europe began investigating an outbreak of Shiga toxin–producing *Escherichia coli* (STEC) O104:H4 infections that ultimately involved more than 4,000 persons in 16 countries. Early in the outbreak, it became evident that international surveillance would be necessary to determine the scope of the outbreak, characterize the disease, and identify the source. This report describes surveillance conducted in the United States, which involved active case-finding, use of laboratory testing protocols specific to non-O157 STEC, interviews to identify potential exposures of interest, and documentation of clinical courses. Six cases in the United States were associated with the outbreak. Although European epidemiologic studies, including analyses of restaurant cohorts and traceback investigations, ultimately implicated raw fenugreek sprouts as the food vehicle, none of the patients in the United States definitively recalled sprout consumption. These events highlight challenges in investigating outbreaks, particularly those caused by rare pathogens or associated with food vehicles that are consumed in small quantities as part of other dishes. Clinical laboratories should adhere to STEC testing recommendations because they are critical for identification of rare or novel STEC pathogens. Robust public health infrastructure is necessary to effectively manage and resolve foodborne outbreaks.

On May 19, 2011, the Robert Koch Institute in Germany was notified of a cluster of three patients with hemolytic uremic syndrome (HUS) admitted to a single hospital in Hamburg (1). Enteroaggregative STEC O104:H4, a rare pathogen to which few human illnesses had been previously attributed (2), was isolated from patient specimens. Illness onsets began in early May, and cases initially were concentrated in northern Germany. Over the next few weeks, case counts mounted rapidly in Germany, and by June 1, approximately 1,534 cases were identified, including 470 (31%) cases complicated by HUS (3). New cases were quickly identified throughout Europe and elsewhere in persons who had recently traveled in Europe. On May 25, German public health authorities made an International Health Regulations notification to the World Health Organization regarding the substantial rise in STEC O104:H4 cases, and the U.S. Department of State notified CDC. Surveillance was conducted in the United States to assist in determining the extent of the outbreak and to identify case clusters that could be investigated for common food or environmental exposures.

Restaurant cohort studies and traceback investigations conducted in Germany implicated mixed raw sprouts from one farm in Germany, and the product was recalled on June 10 (4). On June 24, France reported a cluster of diarrheal illnesses and HUS cases among attendees at an event near Bordeaux. Ten persons had culture-confirmed STEC O104:H4 infection, and seven developed HUS (5). Ill persons reported consuming locally grown raw sprouts, and subsequent European Food Safety Authority traceback investigations identified one lot of fenugreek seeds imported from Egypt as the source of the sprouts responsible for the outbreaks in Germany and France (6).

On May 26, CDC initiated active surveillance for cases in the United States associated with this outbreak. Objectives included 1) identification of any travel and food consumption commonalities among patients, 2) ascertainment of information about clinical courses of patients, and 3) microbiologic characterization of isolates from patient specimens. CDC asked states to report all cases of STEC diarrheal illness or HUS associated with recent travel to Germany. Announcements were sent electronically via CDC’s Foodborne Outbreak Listserv and Epidemic Information Exchange (Epi-X) web communication network. Additionally, a health advisory for clinicians was distributed via CDC’s Health Alert Network. A suspected primary case was defined as HUS or Shiga toxin–positive diarrheal illness in a person who had traveled to Germany after April 1, 2011, and illness onset either during travel in Germany or within 3 weeks after returning from Germany. A suspected secondary case was defined as HUS or diarrheal illness in a person who had not traveled to Germany within 3 weeks of illness, but who had contact with a person with a confirmed case. Cases were confirmed when STEC O104:H4 with a pulsed-field gel electrophoresis (PFGE) pattern matching the outbreak strain was isolated from a clinical specimen.

STEC O104:H4 was isolated at state public health laboratories or at CDC from Shiga toxin–positive stool enrichment broths submitted by clinical laboratories. Isolates were subtyped by PFGE in PulseNet, the national subtyping network for foodborne disease surveillance, and characterized at CDC with serotyping, antimicrobial susceptibility testing, and virulence profiling. Patients were contacted to complete food exposure questionnaires designed and provided by the Robert Koch Institute and to undergo open-ended interviews regarding food consumption and environmental exposures during the 3 weeks before illness onset. In instances where the patient was too ill to undergo interview, travel companions were identified and questioned as proxies. To detect any unusual food items or other exposures in common, a single-interviewer strategy was employed.

What is already known on this topic?

Although Shiga toxin–producing *Escherichia coli* (STEC) O157 is an often identified pathogen, illnesses involving non-O157 serogroups are increasingly recognized. During May–July 2011, a large outbreak of STEC O104:H4 occurred in Europe and North America that was associated with consumption of raw fenugreek sprouts. In addition to producing Shiga toxin, the strain had the characteristics of the enteroaggregative *E. coli* pathotype. This combination of virulence characteristics seems to have increased the pathogenicity of the strain.

What is added by this report?

Surveillance conducted in the United States during the outbreak identified six cases associated with the outbreak; four were complicated by hemolytic uremic syndrome. These are the first documented cases of STEC O104:H4 in the United States. None of the U.S. patients recalled consumption of sprouts, the outbreak vehicle.

What are the implications for public health practice?

Adherence to recommended STEC diagnostic testing is critical in detecting illnesses and outbreaks attributable to non-O157 STEC. High-quality public health infrastructure allows for comprehensive responses and rapid control of outbreaks that might involve similarly rare pathogens.

During May 26–June 16, six confirmed cases were identified in five states: Arizona (one), Massachusetts (one), Michigan (two), North Carolina (one), and Wisconsin (one). Ages of patients ranged from 38 to 72 years (median: 52 years); two patients were female. Five reported travel to or from Germany in the 3 weeks before their illness; the additional secondary case was in a close relative of a patient who had traveled. Patients reported consumption of various fresh produce items while in Germany, such as tomatoes, lettuce, and cucumbers. However, none recalled consumption of sprouts, the food vehicle ultimately implicated in the outbreak. All patients had diarrhea, including four (66%) with bloody diarrhea. Four (66%) patients were reported by physicians to have developed HUS, requiring dialysis and ventilator support. One patient died.

Microbiologic characterization of the pathogens isolated from clinical specimens demonstrated STEC O104:H4 within all specimens, with a PFGE pattern indistinguishable from the outbreak strain. This strain combines features of the STEC and enteroaggregative (EAEC) pathotypes of *Escherichia coli*. All isolates were positive for the stx2a gene, which encodes a Shiga toxin variant often associated with severe *E. coli* illness, such as bloody diarrhea or HUS (7). Additionally, all isolates were positive for the aggR gene, which encodes a regulator of virulence plasmid and chromosomal genes and is characteristic of EAEC (7). The strains were negative for the eae gene, which encodes a mucosal adherence protein in most STEC (7). Isolates from the six patients had almost identical antimicrobial resistance profiles; all were resistant to ampicillin, ceftriaxone, streptomycin, sulfisoxazole, tetracycline, and trimethoprim/sulfamethoxazole.

On July 5, 2011, the European Union banned importation of fenugreek seeds and various other seeds, beans, and sprouts from Egypt. Worldwide surveillance continued for an additional 3 weeks, but no new cases were identified. On July 26, public health authorities in Germany declared the outbreak to be over. The final case count was 4,075 cases (including 908 cases complicated by HUS) and 50 deaths in 16 countries (3).

## Editorial Note

This report summarizes the second-largest STEC outbreak worldwide and includes the first documented STEC O104:H4 illnesses in the United States (1). Although only six cases were identified in the United States, the clinical severity of the illnesses associated with the strain warranted aggressive surveillance. Identifying and investigating these cases allowed for more complete understanding of this uncommon pathogen. Previous surveillance indicates that HUS complicates approximately 6% of STEC O157:H7 infections (1, 2); however, HUS occurred in 66% of U.S. cases and 22% of worldwide cases (including the U.S. cases) during this outbreak, indicating that the outbreak strain might have been especially virulent. Surveillance provided important information regarding disease transmission; one instance of secondary transmission was documented among U.S. cases. Additionally, investigation of these cases reinforced the utility of the recipe-based restaurant analytic studies employed by German investigators. Inability to recall sprout consumption was apparent in U.S. and German patients. In early interviews, only 25% of German HUS patients reported eating sprouts in the 2 weeks before their illness (4). Limited recall for food items consumed as ingredients within dishes has proved challenging in other foodborne outbreak investigations, particularly outbreaks involving raw produce (9). Restaurant-based investigations allow for the identification of a cohort with exposure to a limited menu of dishes and ingredients that can be exhaustively scrutinized for commonalities, addressing the problem of incomplete recall of so-called “stealth vehicles” (9).

This outbreak also highlights the importance of adherence to laboratory testing recommendations in the identification of outbreak pathogens, particularly when the outbreak involves rare or novel pathogens. CDC’s recommended protocols for routine testing of acute community-acquired diarrhea specimens call for assays to detect Shiga toxin, simultaneous culture on selective and differential agar to distinguish STEC O157, which accounts for approximately half of all laboratory-confirmed STEC infections in the United States (10), and additional testing of Shiga toxin–positive specimens at public health laboratories to identify non-O157 STEC (10). Recent evaluations of laboratories in the United States involved in processing diarrhea specimens indicated that 22% of surveyed laboratories adhered to recommendations.* More complete adherence would allow for better detection and identification of both O157 and non-O157 illness-causing STEC. Prompt and accurate pathogen identification and diagnosis is critical for both patient and outbreak management.

Effectively responding to foodborne disease outbreaks, such as the outbreak described in this report, relies on having robust public health infrastructure in place. Such infrastructure, including systematic disease surveillance, laboratory capacity, and the ability to conduct epidemiologic and traceback investigations, is essential for maintaining a safe food supply. Sustaining and enhancing capacity to conduct these activities, both internationally and domestically, will be critical in confronting future challenges related to known and novel pathogens.

## Footnotes

* Additional information available at https://idsa.confex.com/idsa/2012/webprogram/Paper37586.html.

## References

[1] Frank C, Werber D, Cramer JP (2011). Epidemic profile of Shiga-toxin–producing *Escherichia coli* O104: H4 outbreak in Germany. N Engl J Med.

[2] Scavia G, Morabito S, Tozzoli R (2011). Similarity of Shiga toxin-producing *Escherichia coli* O104:H4 strains from Italy and Germany. Emerg Infect Dis.

[3] World Health Organization (2011). EHEC outbreak: increased cases in Germany.

[4] Robert Koch Institute (2011). Technical report: EHEC/HUS O104 outbreak, Germany, May/June 2011.

[5] King LA, Nogareda F, Weill FX (2012). Outbreak of Shiga toxin–producing *Escherichia coli* O104: H4 associated with organic fenugreek sprouts, France, June 2011. Clin Infect Dis.

[6] European Food Safety Authority (2011). Tracing seeds, in particular fenugreek (*Trigonella foenum-graecum*) seeds, in relation to the Shiga toxin-producing *E. coli* STEC O104:H4 outbreaks in Germany and France.

[7] Scheutz F, Møller Nielsen E, Frimodt-Møller J (2011). Characteristics of the enteroaggregative Shiga toxin/verotoxin-producing *Escherichia coli* O104:H4 strain causing the outbreak of haemolytic uraemic syndrome in Germany, May to June 2011. Euro Surveill.

[8] Gould LH, Demma L, Jones TF (2009). Hemolytic uremic syndrome and death in persons with *Escherichia coli* O157: H7 infection, foodborne diseases active surveillance network sites, 2000–2006. Clin Infect Dis.

[9] Barton Behravesh C, Mody RK, Jungk J (2011). 2008 outbreak of *Salmonella* Saintpaul infections associated with raw produce. N Engl J Med.

[10] CDC (2009). Recommendations for diagnosis of Shiga toxin–producing *Escherichia coli* infections by clinical laboratories. MMWR.

